# The Luteinizing Hormone Receptor Knockout Mouse as a Tool to Probe the In Vivo Actions of Gonadotropic Hormones/Receptors in Females

**DOI:** 10.1210/endocr/bqab035

**Published:** 2021-02-19

**Authors:** Kim Carol Jonas, Adolfo Rivero Müller, Olayiwola Oduwole, Hellevi Peltoketo, Ilpo Huhtaniemi

**Affiliations:** 1 Department of Women and Children’s Health, King’s College London, London SE1 1UL, UK; 2 Institute of Reproductive and Developmental Biology, Department of Metabolism, Digestion and Reproduction, Imperial College London, London W12 0NN, UK; 3 Institute for Biomedicine, Department of Physiology, University of Turku, 20520 Turku, Finland; 4 Department of Biochemistry and Molecular Biology, Medical University of Lublin, 20-093 Lublin, Poland; 5 Laboratory of Cancer Genetics and Tumour Biology, Cancer and Translational Medicine Research Unit, Biocenter Oulu and University of Oulu, 90220 Oulu, Finland

**Keywords:** gonadotropin hormones, reproduction, luteinizing hormone, follicle-stimulating hormone, G protein-coupled receptors

## Abstract

Mouse models with altered gonadotropin functions have provided invaluable insight into the functions of these hormones/receptors. Here we describe the repurposing of the infertile and hypogonadal luteinizing hormone receptor (LHR) knockout mouse model (LuRKO), to address outstanding questions in reproductive physiology. Using crossbreeding strategies and physiological and histological analyses, we first addressed the physiological relevance of forced LHR homomerization in female mice using BAC expression of 2 ligand-binding and signaling deficient mutant LHR, respectively, that have previously shown to undergo functional complementation and rescue the hypogonadal phenotype of male LuRKO mice. In female LuRKO mice, coexpression of signaling and binding deficient LHR mutants failed to rescue the hypogonadal and anovulatory phenotype. This was apparently due to the low-level expression of the 2 mutant LHR and potential lack of luteinizing hormone (LH)/LHR-dependent pleiotropic signaling that has previously been shown at high receptor densities to be essential for ovulation. Next, we utilized a mouse model overexpressing human chorionic gonadotropin (hCG) with increased circulating “LH/hCG”-like bioactivity to ~40 fold higher than WT females, to determine if high circulating hCG in the LuRKO background could reveal putative LHR-independent actions. No effects were found, thus, suggesting that LH/hCG mediate their gonadal and non-gonadal effects solely via LHR. Finally, targeted expression of a constitutively active follicle stimulating hormone receptor (FSHR) progressed antral follicles to preovulatory follicles and displayed phenotypic markers of enhanced estrogenic activity but failed to induce ovulation in LuRKO mice. This study highlights the critical importance and precise control of functional LHR and FSHR for mediating ovarian functions and of the potential repurposing of existing genetically modified mouse models in answering outstanding questions in reproductive physiology.

The coordinated actions of the gonadotropic hormones, luteinizing hormone (LH) and follicle-stimulating hormone (FSH) and their cognate receptors are essential for reproduction (1, 2). Rare naturally occurring mutations in humans, and laboratory-generated mutations in genetically modified mice, as well as in vitro analyses, have provided vital information on structure–function pairing for gonadotropin hormone/receptor activation, trafficking, and signaling (3-7). In particular, in vivo studies from animal models have proved to be powerful tools for studying the intricacies of gonadotropic hormone/receptor function in a physiologically relevant manner, elucidating their roles in the female in follicle recruitment, selection and growth, ovulation, and corpus luteum function (8-11). As such, these genetically modified animal models have revealed many important nuances in the molecular and physiological control of reproduction.

A key genetically modified mouse model that has provided important insights into the physiological roles of the luteinizing hormone receptor (LHR), is the LHR knockout (LuRKO) mouse (10). LuRKO animals of both sexes present phenotypically with pubertal delay, hypogonadism, and infertility, and have revealed the roles of LHR in pubertal attainment and maintenance of fertility in adulthood (10). Furthermore, LHR function was found redundant for the prenatal sexual differentiation and maturation of both sexes (10). Studies, by us and others, have challenged the central dogmas in the role of LHR in males and females. We therefore aimed to repurpose and utilize existing previously characterized mouse models of gonadotropin/receptor modifications to address several outstanding questions surrounding the molecular mechanisms by which the LHR and its ligands mediate their physiological functions in female mice. By utilizing the LuRKO mouse as a background phenotype to be crossed with 3 additional genetically modified mouse models, we first show that female bacterial artificial chromosome (BAC) transgenic expression of transactivating mutant LHRs, previously shown to undergo intermolecular cooperation in vitro and in vivo (12-14), failed to alter the infertile female LuRKO phenotype. Second, that transgenic over-expression of the highly active LHR ligand, human chorionic gonadotropin (hCG) (15), proposed to have LHR independent actions (16, 17), had no such effects on the hypogonadal phenotype in LuRKO females. Third, expression of constitutively activating mutant (CAM) follicle stimulating hormone receptor (FSHR) (18) was capable of increasing the ovarian follicle development in absence of LHR but failed to induce ovulation and rescue fertility in females, contrasting with FSH treated hypophysectomized female mice (19) and male LuRKO mice expressing CAM FSHR (20). These data highlight the critical importance of LHR in maintenance of female ovarian function and cyclicity.

## Materials and Methods

### Animals

LuRKO mice were produced by targeted disruption of exon 11 of *Lhr* as previously described (10). Transgenic mice expressing either signal- (LHR^S–^) or binding-deficient (LHR^B–^) form of the Lhr, generated by inserting mutated bacterial artificial chromosomes (BACs), were crossed with LuRKO heterozygotes for 2 generations to obtain LuRKO^–/–^ carrying either 1 or both transgenes, as described previously (12). hCG beta transgenic mice were generated using the *ubiquitin C* promoter to drive ubiquitous expression of *hCG beta* transgene as previously described (15). To generate the double transgenic line with hCG beta expression in a LuRKO background, heterozygous LuRKO animals were intercrossed with hCG beta expressing mice. The resulting hCG beta/heterozygous LuRKO^–/+^ mice were subsequently intercrossed to produce hCG beta/LuRKO double transgenic lines. The CAM-FSHR mice were generated by expressing *Fshr*-D580H under the human *anti-Müllerian hormone* promoter (18). The double mutant *Fshr*-CAM mice on a homozygous *Lhr*^*–/–*^ background (LuRKO/CAM FSHR) were generated using a 3-step breeding program as previously described (20). Briefly, the *Lhr*^*+/–*^ females were first backcrossed into FVB/N background, and the females produced were intercrossed with the male transgenic *Fshr*-CAM mice to obtain the *Fshr*-CAM/*Lhr*^+/–^ males, which were finally crossbred with the *Lhr*^+/–^ females in FVB/N background. In this context WT mice referred to mice that after the breeding program did not contain the *Fshr*-D580H transgene and expressed *Lhr*. In the analyses *Lhr*^+/–^ mice, showing no phenotype deviant from WT, have been pooled together with WT mice and likewise *Fshr*-CAM/*Lhr*^+/–^ mice with *Fshr*-CAM/*Lhr*^+/+^ mice.

All procedures were carried out in accordance to the regulations of the UK Home Office Animals (Scientific Procedures) Act, the Imperial College London guidelines for animal care, and University of Turku Ethical Committee on Use and Care of Animals approval. To ensure transgene transmission/deletion, ear notches were obtained and genotyped as previously described (10, 12, 15, 18, 20). To assess for pubertal onset, vaginal opening was monitored daily from day 21 until detected, as previously described (18). Vaginal smears were taken daily from the day of vaginal opening for 14 to 21 days, to monitor estrous cyclicity.

### Histological Analyses

Ovaries and uteri were dissected, weighed, and visualized for changes prior to fixation in 4% paraformaldehyde for 4 to 24 hours depending on size, or snap frozen in liquid nitrogen for gene expression analysis. Fixed tissues were dehydrated in graded ethanol solutions until absolute water-free, cleared in histoclear (National Diagnostics, Hessle Hull, UK) and embedded in paraffin. Ovaries were serially sectioned at 5 μm thickness, mounted on polylysine slides (VWR, Lutterworth, UK), dried at 37°C for approximately 1 hour, and stored for subsequent use. For histological analysis, tissue sections were stained with the standard hematoxylin and eosin protocol. To assess the presence of corpora lutea and follicle morphology every 10th to 15th section was imaged, with the presence or absence of corpora lutea and cumulus oocyte complex expansion recorded. For concordance in image size, representative images were taken from the middle cross-section of ovaries. Vaginal cytology samples were taken daily from the first day of vaginal opening and stained by the Giemsa method. Estrous stages were defined as proestrous, estrous, metestrous, and diestrous and transitions from 1 stage to another as previously described (21). Mammary gland tissue samples were collected, whole-mounted, and stained with Carmine Alum, as previously described (15). The presence or absence of ductal elongation reaching the lymph node was observed from 3 to 5 age-matched females in each genotype group (WT and LuRKO, n = 3; CAM-FSHR, n = 4; LuRKO/CAM-FSHR, n = 5). All histological samples were imaged using a Nikon Eclipse ME600 with a mounted Nikon D1500 digital camera.

### Measurement of Serum LH

For collection of serum for LH measurement, mice were euthanized using a terminal dose of Avertin and blood collected by cardiac puncture. Serum LH was measured by immunofluorometric assay, as previously described (22).

### Quantitative Real-time Polymerase Chain Reaction

Total mRNA was extracted from ovaries and purified using TRIsure and phenol-chloroform following clean-up with RNeasy kit (Qiagen), including DNase treatment. The purity and quantity of isolated RNA was estimated spectrophotometrically with the use of Nanodrop (ThermoFisher). Reverse transcription (RT) was performed with AMV-reverse transcriptase (Promega). Quantitative polymerase chain reaction (qPCR) reactions were performed using DyNAmo SYBR Green (Finnzymes) kit. PCR reactions were performed in triplicate in a qPCR thermocycler (Chromo4 with OpticonMonitor software, Bio-Rad), using specific primers to *Lhrs* (Wt, LHR^B–^ or LHR^S–^), *Cyp11a1, Cyp19a1* and corresponding housekeeping genes (Table 1).

**Table 1.** qPCR primers utilized for assessment of ovarian transcripts

qRT-PCR primer	Forward sequence	Reverse sequence
*Total mLHR*	AGCATCTGTAACACAGGCATCC	CACAGCGTGATGGACTCATTAT
*mLHR* ^*B–*^	GCAGCACGACTTCTTCAAGTCCGCCATGCC	GTGGCGGATCTTGAAGTTGGCCTTGATGCC
*mLHR* ^*S–*^	GTGATGCAGAAGAAGACCATGGGCTGGGA	ATGTCCAGCTTGGCGTCCACGTAGTAGTAG
*Ppia*	CATCCTAAAGCATACAGGTCCTG	TCCATGGCTTCCACAATGTT
*Gapdh*	AGGTCGGTGTGAACGGATTTG	GGGGTCGTTGATGGCAACA
*Actb*	CGTGGGCCGCCCTAGGCACCA	TTGGCCTTAGGGTTCAGGGGG
*Cyp11a1*	AGATCCCTTCCCCTGGTGACAATG	CGCATGAGAAGAGTATCGACGCATC
*StAR*	CAGGGAGAGGTGGCTATGCA	CCGTGTCTTTTCCAATCCTCTG
*Cyp19a1*	CGGGCTACGTGGATGTGTT	GAGCTTGCCAGGCGTTAAAG
*Prlr*	CTGGTTGGTTTACAATGGAA	AACGACTGGCCCAGAGGCTCCCTG
*Hsd17b3*	CGGGAAAGCCTATTCATTTG	TCACACAGCTTCCAGTGGTC
*Cyp17a1*	CGTCTTTCAATGACCGGACT	CATAAACCGATCTGGCTGGT

A linear standard curve was drawn using different dilutions of a plasmid containing the cDNA of the *Lhr*. Results were adjusted to the housekeeping gene expression. Quantification of gene expression in different mouse strains was normalized to 2 housekeeping genes (*Gapdh* and *ActB*). At least 4 samples per group were analyzed in at least duplicates. Primers for qRT-PCR are shown in Table 1.

### Statistical Analysis

Statistical analyses were performed in GraphPad Prism, V8 using 1-way analysis of variance (ANOVA) with Dunnett’s multiple comparison post-hoc testing. Data is represented as mean ± SEM for n = 4 to 15 animals. Statistical significance was determined as a *P* < .05.

## Results and Discussion

### In Vivo LHR Homomerization via Functional Complementation in Female Mice

**Figure 1.**
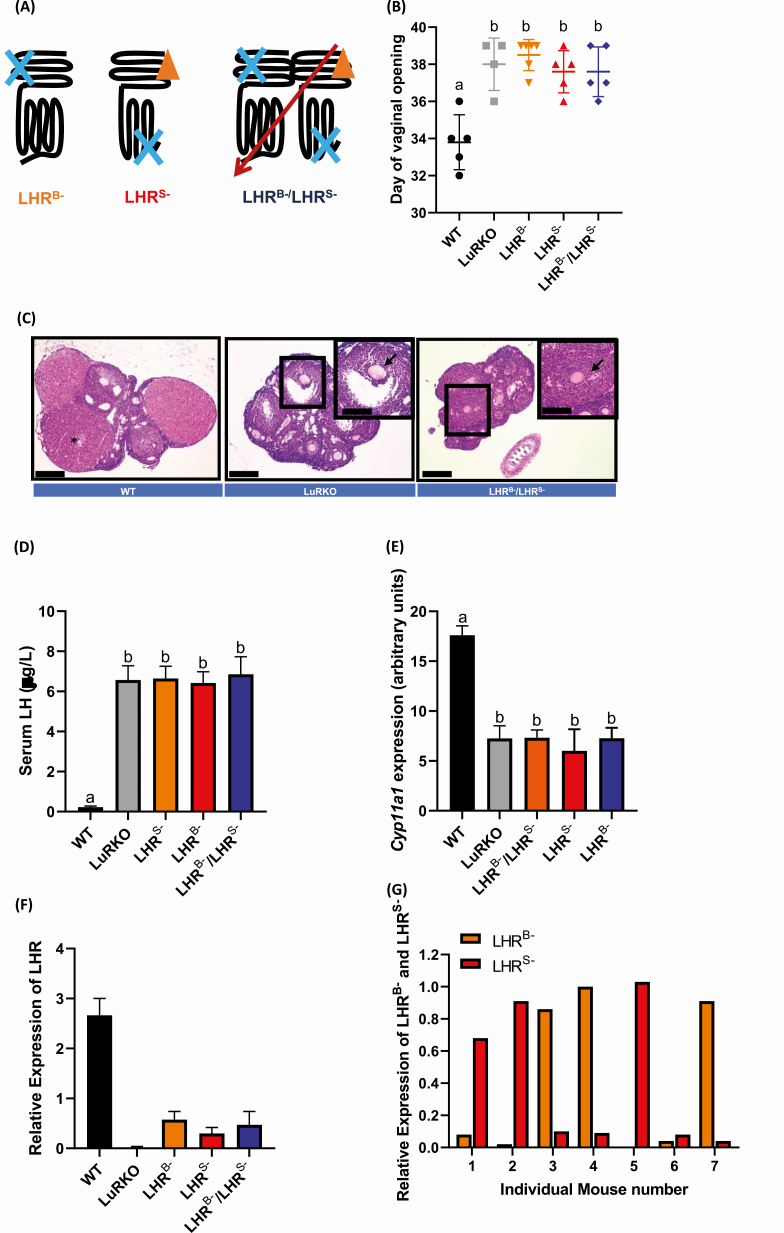
LHR functional complementation has no effect on the hypogonadal phenotype of female LuRKO animals due to low LHR^B–^/LHR^S–^ expression. (A) Schematic detailing LHR functional complementation and forced homomerization using LHR^B–^ and LHR^S–^ mutant receptors. The effects of LHR^B–^ and LHR^S–^ BACs co-expressed in a LuRKO background on (B) day of vaginal opening. Statistical significance determined by 1-way ANOVA, a versus b = *P* < .001, WT versus LuRKO animals, with no significant difference between LuRKO, LHR^B–^, LHR^S–^ and LHR^B–^/LHR^S–^ groups (n = 4 for LuRKO, n = 5 for WT, LHR^S–^ and LHR^S–^/LHR^B–^, n = 6 for LHR^B–^). (C) Representative ovarian histology in 3-month-old control WT, LuRKO alone, or a LuRKO background expressing LHR^B–^/LHR^S–^ female littermates. *Presence of corpora lutea, arrows indicate failure of cumulus-oocyte-complex expansion. (D) Analysis of serum LH from WT, LuRKO, LURKO/LHR^B–^, LuRKO/LHR^B–^ and LuRKO coexpressing LHR^B–^/LHR^S–^. Statistical significance was determined by 1-way ANOVA with Dunnett’s multiple comparisons to the control and between groups. Alphabetic denotation a versus b = *P* < .0001, WT versus LuRKO animals, with no significant difference between LuRKO, LHR^B–^, LHR^S–^ and LHR^B–^/LHR^S–^ groups (n = 6 animals per group). Relative transcript levels of (E) *P450scc* and (F) *Lhr* from ovarian extracts of female WT, LuRKO, LuRKO/LHR^B–^, LuRKO/LHR^S–^ and LuRKO/LHR^B–^/LHR^S–^ (n = 8 for LuRKO, LHR^B–^ and LHR^S–^; n = 10 for WT; and n = 12 for LHR^B–^/LHR^S–^). (G) relative transcript levels of *Lhr*^*B–*^ and *Lhr*^*S–*^ in single female mice co-expressing *Lhr*^*B–*^/*Lhr*^*S*–^. Statistical significance in (E) was determined by 1-way ANOVA with Dunnett’s multiple comparisons to the control and between groups. Alphabetic denotation a versus b = *P* < .0001, WT versus LuRKO animals, with no significant difference between LuRKO, LHR^B–^, LHR^S–^ and LHR^B–^/LHR^S–^ groups.

There is increasing evidence that G protein-coupled receptor di/oligomerization provides an important means to diversify/bias receptor functions (23, 24). Our previous study showed that “forced” LHR homomerization via functional complementation was sufficient to restore the Leydig cell testosterone production and fertility of LuRKO male mice (12). However, whether LHR homomerization could restore the ovarian functions and fertility of female LuRKO remained unknown. Here, using the same BACs transgenic approach as previously employed, and functional complementation of binding (LHR^B–^) and signaling deficient (LHR^S–^) mutant LHRs (Fig. 1A) we analyzed the effect of LHR^S–^ and LHR^B–^ co-expression in the LuRKO background on key reproductive parameters to assess this question.

Because the postnatal sexual maturation is impaired in LuRKO animals, we first examined if LHR^B–^/LHR^S–^ coexpression could alter the timing of puberty via monitoring the day of vaginal opening. In wild-type (WT) and heterozygous LuRKO control animals, the first day of vaginal opening occurred at 33.8 ± 0.7 days (Fig. 1B). In contrast, in LuRKO/LHR^B–^/LHR^S–^ females, the onset of puberty was delayed to 37.6 ± 0.6 days, mirroring the pubertal delay of 38.0 ± 0.7 days, observed in the LuRKO animals (Fig. 1B). Single expression of either LHR^B–^ or LHR^S–^ in the LuRKO background also had no effect on the pubertal delay observed in the LuRKO animals, suggesting that functional complementation failed to rescue the pubertal delay observed in female LuRKO animals, thus highlighting the essential role of LHR in gonadarche.

To determine if coexpression of LHR^B–^/LHR^S–^ could rescue the anovulatory phenotype of the adult LuRKO female mice, ovaries from 3- to 4-month-old female mice were serially sectioned and histologically analyzed. The ovaries from the control heterozygous LuRKO and WT animals showed follicles of all stages of folliculogenesis and the presence of several corpora lutea (Fig. 1C), indicating that ovulation had occurred. In contrast, in the LuRKO females coexpressing the LHR^B–^/LHR^S–^, although primordial, preantral, and antral follicles were present, folliculogenesis was arrested at the large antral to preovulatory follicle stage of development (Fig. 1C). Moreover, serial sectioning of the ovaries failed to locate the presence of any corpora lutea, indicating that these large antral follicles failed to undergo ovulation, as observed in the LuRKO animals (Fig. 1C) (10). Measurement of serum LH supported the hypogonadal phenotype observed in the LURKO females coexpressing LHR^B–^/LHR^S^ (Fig. 1D), with LH significantly elevated compared with control, suggesting an increased hypothalamic–pituitary drive and lack of ovarian steroid hormone feedback. This was in concordance with observations in the LuRKO females in the presence of absence of single LHR^B–^ or LHR^S–^ expression. Analysis of the LH-responsive gene and key steroidogenic enzyme, *Cyp11a1*, also supported diminished steroid hormone production, with significantly decreased *Cyp11a1* expression observed in LURKO females co-expressing the LHR^B–^/LHR^S^ versus WT animals (7.3 ± 0.23 LuRKO/ LHR^B–^/LHR^S^ versus 17.6 ± 0.33 WT, *P* < .0001, Fig. 1E) and no difference when compared with LuRKO mice. Over the study period, >25 female LuRKO coexpressing LHR^B–^/LHR^S–^ mice were cohoused with male LuRKO/ LHR^B–^/LHR^S–^ or WT males with proven fertility. The female mice failed to present with vaginal plugs, nor was a single pregnancy detected, even following superovulation treatment (see (26)). Analysis of key ovarian LH-responsive genes post superovulation supported the anovulatory infertility of the LuRKO female mice coexpressing LHR^B–^/LHR^S–^; with superovulation increasing StAR and prolactin receptor expression in WT animals, but neither were induced in LuRKO nor LuRKO mice co-expressing LHR^B–^/LHR^S^ (Fig. 1 (26)).

The regulation of LHR expression is much more dynamic in the ovary than testis, requiring induced expression in granulosa cells in the mature large antral follicle (10, 27, 28), the activation of multiple G protein-dependent pathways (29, 30) and transactivation of the epidermal growth factor receptor (31-33) for initiating key ovulatory pathway networks. We therefore wanted to interrogate if the expression levels of LHR^B–^ and LHR^S–^ were similar to the WT LHR and whether this could account for disparity between the functional rescue observed between the male versus female mice. qPCR analysis revealed that the combined expression levels of LHR^B–^ and LHR^S–^ in the LuRKO/LHR^B–^/LHR^S–^ animals were just 20% of the WT LHR (Fig. 1F). The expression levels of LHR^B–^ and LHR^S–^ were therefore most likely insufficient to mediate the functional rescue, possibly due to lack of intact mechanisms to trigger LHR upregulation. Granulosa cell expression is required for coupling to Gαq and facilitating FSHR cross talk/heteromerization and epidermal growth factor receptor transactivation necessary for progression of ovulation and high expression levels of LHR have been shown to be necessary for Gαq coupling (34). Additionally, our previous in vitro data suggest that although Gαs activation is intact in WT and LHR^B–^/LHR^S–^ co-expressing conditions, LH (but not hCG)-dependent Gαq coupling is diminished in the latter (14). Therefore, the lack of ovulation observed in LuRKO mice coexpressing LHR^B–^/LHR^S–^ may reflect an impaired receptor density and ability to activate LH-dependent Gαq signaling.

Our previous results have shown that the ratio of LHR^B–^ to LHR^S–^ directs the amplitude of Gαs and Gαq signaling observed (14). We therefore determined the levels of LHR^B–^ and LHR^S–^ in individual mice coexpressing LHR^B–^/LHR^S–^. Surprisingly, we saw a wide range of expression levels, ranging from 1:22 LHR^B–^:LHR^S–^ to 45:1 LHR^B–^: LHR^S–^ (Fig. 1G). Yet, in all cases the expression of individual or combined *Lhr* expression (by qPCR) was only a fraction of that of the WT in control animals (<25%). Our previous reported in vitro analysis of the ratiometric effects of LHR^B–^:LHR^S–^ expression suggested that an excess of cell surface LHR^S–^:LHR^B–^ promoted more effective Gαs and Gαq signaling, contrasting with this *in vivo* data. However, the combined cell surface expression levels of LHR^B–^ and LHR^S–^ in cell lines analyzed were comparable to WT LHR, which may account for the disparity between our previous in vitro findings, and this study.

Overall, physiologically, these data suggest a much simpler male regulation of LHR functions, with a small amount of LHR signaling (<1% receptor occupancy) sufficient to trigger testosterone generation, in concordance with previous studies (35) and therefore rescuing spermatogenesis, as compared to the cyclical changes and more complex LHR actions evoked by higher LH levels and receptor density and occupancy in females. Additionally, while cellular compartmentalized expression of LHR and the FSHR occurs in males, in females LHR and FSHR are coexpressed in antral follicles of granulosa cells, which is hypothesized to be essential for the latter stages of ovarian follicle maturation and ovulation, via LHR-Gαq activation (30). If such multifaceted LHR-dependent signaling activities are required in females (vs males) is a question that remains unanswered. However, with the advent of CRISPR, in all its forms, and thus the technologies to gene edit and regulate gene expression, it is conceivable to envision such experiments and again repurpose the LuRKO mice.

### The LH Agonist hCG Acts Explicitly via LHR

**Figure 2.**
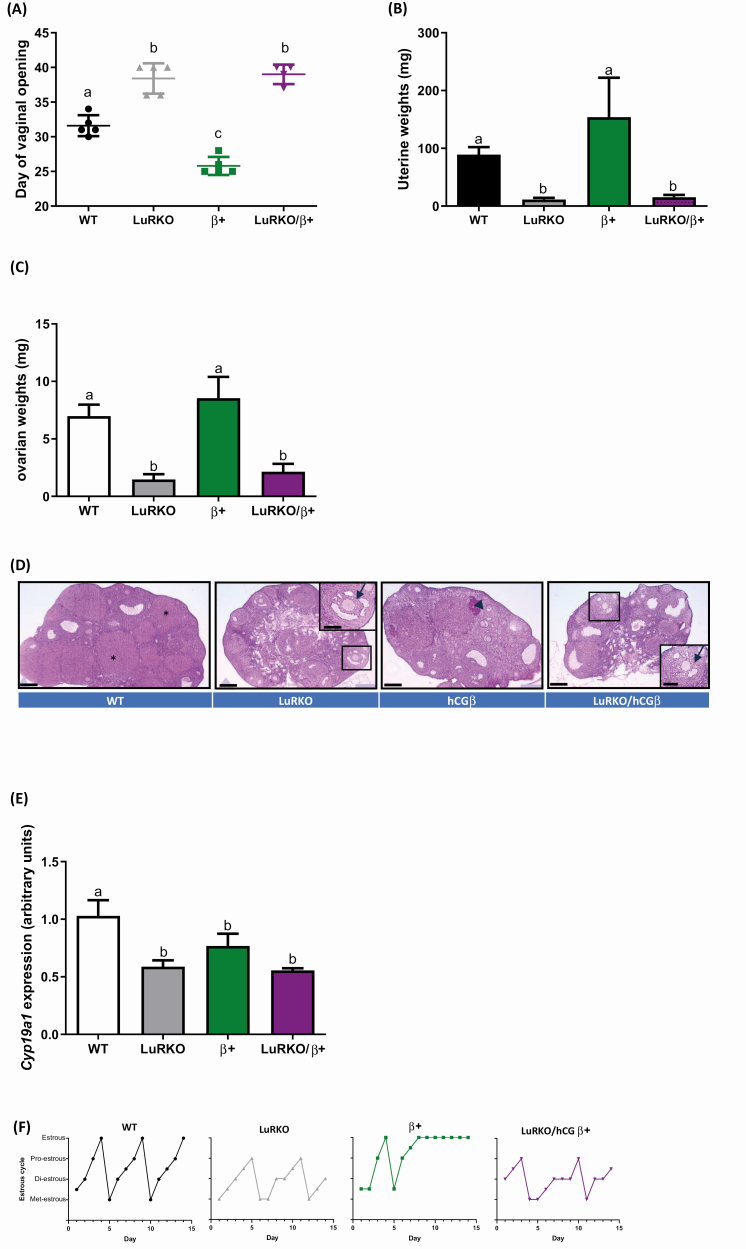
Enhanced “LH-like” activity via hCG fails to rescue the hypogonadal phenotype of female LuRKO mice. The effects of hCG overexpression in the absence of LHR on key reproductive parameters as assessed by (A) day of vaginal opening checked daily from d21 weaning and (B) Uterine weights in WT, LuRKO, hCG beta (β) and LuRKO/hCG beta animals (LuRKO/β) females. Statistical analysis via 1-way ANOVA with Dunnett’s multiple comparisons to the control and between groups. N Differential letter denotation equaled statistical significance between experimental groups, with a vs b = *P* < .0001, a vs c = *P* < .001, and b vs c = *P* < .0001. n = 5 for WT, LuRKO, and hCG beta groups, and n = 4 for LuRKO/hCG beta mice. (C) Ovarian weights showing hypogonadal phenotype of LuRKO/hCG beta females, 1-way ANOVA with Dunnett’s multiple comparisons analysis was conducted comparing between all groups. Differential letter denotation equaled statistical significance between experimental groups, with a versus b = *P* < .05. n = 5 for WT, LuRKO, and hCG beta groups, and n = 4 for LuRKO/hCG beta mice. (D) histological analysis of ovarian sections, with representative ovarian sections taken from the central part of the ovary with multiple large antral follicles displayed. The ovaries of LuRKO/hCG beta mice were comparable to LuRKO animals, with follicles arrested at the large antral phase (enlarged image inset) lacking cumulus oocyte complex expansion, and absence of corpora lutea (*example corpus luteum in WT animals). For the hCG beta mice, a hemorrhagic cyst, typical for mice with hCG beta expression, was also observed (arrowhead). Scale bars = 200 µm, with inset 400 µm. WT included *Lhr*^*+/–*^ females. Representative from n = 4 for all experimental groups. (E) Relative expression of *Cyp19a1*. Statistical analysis via One-way ANOVA with Dunnett’s multiple comparisons analysis, with a versus b, *P* < .05. n = 4 for all experimental groups (F) Representative examples of estrous cycles of each experimental group. Staging was Met, metestrous; Di, diestrous; Pro, proestrous; Estr, estrous. Transition stages have been marked between the 2 stages. n = 4 for all experimental groups.

Recent studies have suggested that LHR agonists LH and hCG may mediate its effects via alternative receptors to the LHR (16, 17, 36). We therefore utilized our previously described hCG beta overexpressing transgenic mouse line, with circulating bioactive heterodimeric hCG concentrations approximately 40-fold higher than endogenous LH levels typically found in WT animals (15). These mice were crossed into the LuRKO mouse background to determine whether high LH/hCG bioactivity could have any effects on the phenotype of the animals devoid of functional LHR. Assessment of postnatal sexual development showed a delay in the first day of vaginal opening of the LuRKO/hCG beta mice (Fig. 2A) comparison with WT (39.2 ± 2.1 days LuRKO/hCG beta versus 31.6 ± 1.5 days WT, *P* < .0001), mimicking the delay observed in LuRKO animals. This contrasted with the accelerated onset and precocious puberty observed with the hCG beta mice ((15) and Fig. 2A), therefore, showing the essential role of LHR in post-natal sexual development. Analysis of the uterine weights of LuRKO/hCG beta mice revealed a significant decrease in comparison to control and hCGβ littermates (Fig. 2B), suggestive of estrogen deficiency in the LuRKO/hCG beta animals. The ovarian weights supported a hypogonadal phenotype of the LuRKO/hCG beta mice, which were significantly lower than WT littermates (Fig. 2C, LuRKO/hCG beta 2.15 mg ± 0.67 versus WT, 6.98 mg ± 0.99, *P* < 0.05), but similar to LuRKO females. Histological analysis of serially sectioned ovarian tissue revealed that the LuRKO/hCG beta ovaries contained follicles that were halted at the large antral follicle stage, with absent corpora lutea. This indicated a failure of ovulation, in concordance with the LuRKO animal ovarian phenotype (Fig. 2D) (10), consistent with the essential role of intact LHR signaling for driving ovulation. Assessment of the key steroidogenic enzyme expression, CYP19A1 revealed diminished expression in LuRKO/hCG beta females in comparison to control (Fig. 2E). However, similar expression to LuRKO animals was observed supporting the suggested decrease in estradiol production in the LuRKO/hCG beta females. Additionally, daily monitoring of the estrous cycle revealed that the LuRKO/hCG beta were acyclic (Fig. 2F and Table 2). Interestingly, proestrous was observed in both the LuRKO and LuRKO/hCG beta mice (Fig. 2F and Table 2), suggesting a degree of follicular maturation, as evidenced by the ovarian morphology data (Fig. 2D). However, the mice failed to undergo estrous, mimicking the acyclicity and ovulation failure observed in the LuRKO female littermates, underpinning the importance of LHR for estrous cyclicity.

**Table 2.** The effect of enhanced LH-like activity via hCG on the length of the estrous cycle and time in estrous

Genotype	Number of mice	Days in estrus (mean ± SD)	Length of estrus cycle in days (mean ± SD)
WT	4	1.25 ± 0.5^a^	4.75 ± 0.5
LuRKO	4	0^b^	N/A
hCGb+	4	5.25 ± 2.1^c^	N/A
LuRKO/hCGb+	4	0^b^	N/A

Data comparing mean number of days in estrous and mean cycle length. Data represent mean ± SD of data collected from 4 mice from each experimental group, 2 weeks after first day of vaginal opening. Statistical analysis was via 1-way ANOVA with Dunnett’s multiple comparison post hoc analysis. Statistical difference denoted by different letters, with a versus b *P* < .01, a versus c *P* < .01, b versus c *P* < .0001. N/A represents mice that were acyclic therefore cycle length could not be calculated

These data provide no phenotypic evidence to suggest that high LH/hCG bioactivity can bypass LHR activation in animals devoid of functional LHR. Interestingly, analysis of male LuRKO mice with high circulating hCG also mirrored the infertile and hypogonadal phenotype observed in female LuRKO/hCG beta mice (Fig. 2 (26)), supporting the importance of LHR in maintenance of both male and female gonadal function. Although our data using these mouse models cannot fully address whether in humans alternative functions for hCG exists, such as mannose-6-phosphate receptor–dependent activation in uterine natural killer cells (17) or additional extragonadal roles proposed for hCG (reviewed in (37), it supports that the largest and most important actions of LH/hCG bioactivity are via the gonadal LHR, with most other effects observed downstream of LHR binding and signaling pathway activation. This is further supported by human inactivating or activating LHR mutations, where all the phenotypical abnormalities are connected to alterations in gonadal function (38-43), and also evidenced by fertility achieved through oocyte donation (44). Because LHR inactivation in the LuRKO mice is universal, our studies were not able to address the question about the functional significance of the extragonadal LHR expression (recently reviewed in (45)).

### CAMs of FSHR Partially Rescue the LuRKO Female Phenotype but not Ovulation and Fertility

**Figure 3.**
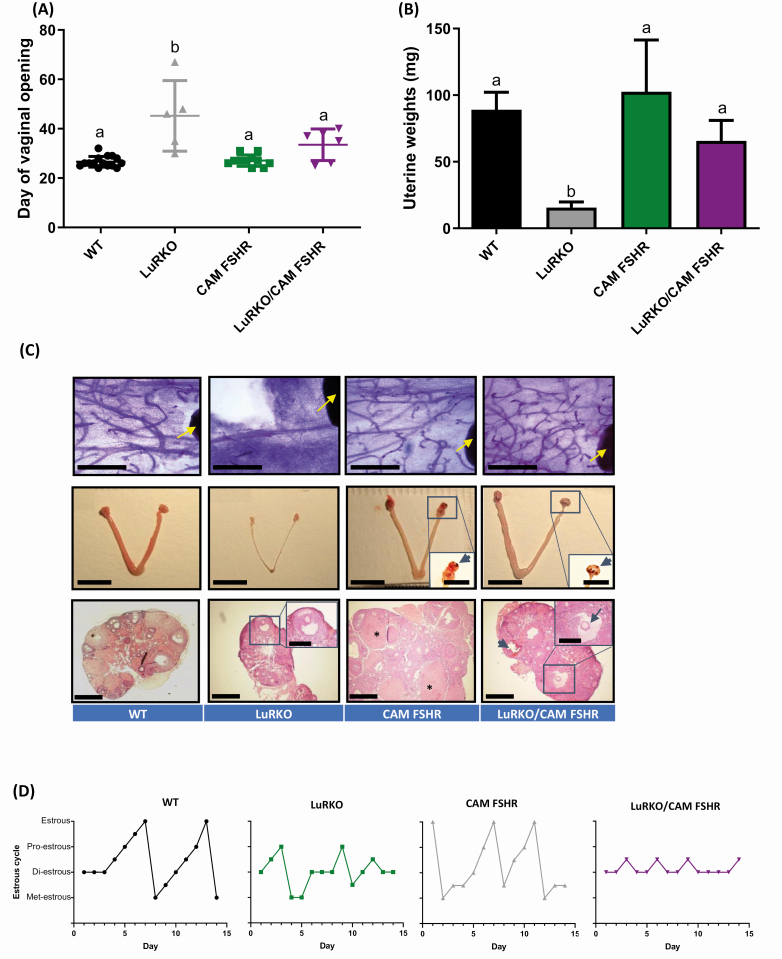
Constitutive activation of FSHR in the absence of LHR partially restores ovarian function and mammary gland development. Key reproductive parameters were assessed via (A) day of vaginal opening and (B) uterine weights in WT, LuRKO /CAM FSHR, CAM FSHR, and LuRKO females. One-way ANOVA conducted using log conversion of uterine weights. a versus b = *P* < .01. For WT, LuRKO and LuRKO/CAM-FSHR groups, n = 3, for CAM-FSHR group n = 4. (C) Representative images of the mammary gland tissue, internal reproductive tracts and ovaries. Histological characterization of mammary gland wholemounts (upper panel) with a reference point lymph node depicted by yellow arrow toward which ductal elongation occurs demonstrating the rescue of elongation in LuRKO/CAM FSHR female mice, versus rudimentary development in LuRKO females. Macroscopic images (middle panel) showing LuRKO females have thin threadlike uteri, while uteri of LuRKO/CAM FSHR mice resemble that of WT littermates. CAM FSHR expression typically results in hemorrhagic cysts in WT and also in LuRKO background (arrowheads in inserts, the backgrounds has been brightened for clarity). Representative ovarian images (lower panel) from central ovarian cross-sections, with the most advanced follicles in the sample presented. The ovaries of CAM FSHR mice were usually larger than those of WT mice and contain multiple developing follicles and luteinizing follicles (*). The ovaries of LuRKO/CAM FSHR mice have more advanced follicles (an arrow in the inset) than LuRKO mice but lack corpora lutea. A hemorrhagic cyst is marked (arrowhead). Scale bars: the uppermost row 1 mm; middle row 10 mm and inserts 50 mm: lowermost row 500 µm and inserts 250 µm. WT included *Lhr*^*+/–*^ females and CAM FSHR in WT and *Lhr*^*+/–*^ backgrounds. (D) Representative examples of estrous cycles. Met, metestrous; Di, diestrous; Pro, proestrous; Estr, estrous. Transition stages have been marked between the 2 stages. CAM FSHR females demonstrated variable cycles from seminormal to acyclic, in line with our previous publication (18), of which 1 has been shown here. For smear analyses WT, n = 8; LuRKO, n = 4; CAM-FSHR, n = 13; LuRKO/CAM-FSHR, n = 6.

Our recent study has shown that the expression of CAM FSHR could rescue the hypogonadal phenotype and infertility of the male LuRKO mice (20), suggesting a role for robust FSHR activity in compensating for missing LHR- and testosterone-dependent gonadal functions, including spermatogenesis. To address whether functional rescue could also take place in the female LuRKO/CAM FSHR littermates, we assessed their key reproductive parameters. Analysis of the day of vaginal opening showed the LuRKO/CAM FSHR mice exhibited more variation in maturation timing, but it was not significantly different from WT females (33.5 ± 6.4 days vs 26.6 ± 2.2 days, respectively), while the delay was considerable in LuRKO female litter mates (45.4 ± 14.3 days, *P* < .05 between LuRKO and LuRKO/CAM FSHR mice) (Fig. 3A). Augmented uterine weights in the LuRKO/CAM FSHR females were also observed in comparison to the LuRKO animals (Fig. 3B). This was also supported by macroanalysis of the uteri (Fig. 3C, middle panel), showing evidence of uterine proliferation in the LuRKO/CAM FSHR mice and suggestive of enhanced estrogen production, whilst the LuRKO animals had thin uteri. The mammary gland elongation in LuRKO/CAM FSHR females provided further indirect evidence of increased estradiol action in comparison to LuRKO females, that were devoid of notable elongation (Fig. 3C, uppermost panel, and (46)). In agreement with the previous data, histological analysis of ovarian tissue showed more advanced antral follicles in the LuRKO/CAM FSHR females, in comparison to LuRKO animals, with clear separation of the cumulus–oocyte complex (Fig. 3C, the lowest panel, an arrow in the insert). This suggests the potential further progression of large antral follicles in the presence of CAM FSHR, and enhanced estrogen production as a result of this. However, ovulation was still absent as no corpora lutea were observed in LuRKO/CAM FSHR ovaries when serially sectioned, contrasting to CAM FSHR females that had often luteinized unruptured follicles, in addition to corpora lutea with trapped oocytes (Fig. 3C and (18)). As a result, the LuRKO/CAM FSHR females were acyclic (Fig. 3D and Table 3), mirroring the arrested cyclicity and lack of estrous observed in LuRKO female mice, showing failure to undergo ovulation. This differed with the CAM FSHR mice, which underwent estrous and occasional ovulation, as previously described (18). As expected, the LuRKO/CAM FSHR females set for breeding with fertile males failed to present with neither vaginal plugs, indicative of the lack of copulatory behavior in the females, nor with pregnancies (data not shown). As with the LuRKO/hCG beta mice, LuRKO/CAM FSHR females presented with variable degrees of follicle development as evidenced by the ovarian histology and estrous cycle data (Fig. 3D, Table 3). Though clear complete estrous cyclicity was not seen in absence of LHR, the advancement from diestrous toward proestrous indicated that follicular growth had been triggered. Follicle growth and estradiol production did not, however, reach sufficient levels to induce estrous.

**Table 3.** The effect of constitutive FSHR activity on the length of the estrous cycle and time in estrous

Genotype	Number of mice	Days in estrus (mean ± SD)	Length of estrus cycle in days (mean ± SD)
WT	8	1.71 ± 0.27^a^	6.2 ± 0.46^a^
LuRKO	4	0 ^b^	N/A
CAM FSHR	13	1.23 ± 0.71^a^	6.9 ± 1.1 or 0^a^
LuRKO/CAM FSHR	6	0^b^	N/A

The mean number of days in estrous and mean cycle length were calculated with data representing the mean ± SD from n = 8 WT; n = 4 LuRKO; n = 13 CAM-FSHR; n = 6 LuRKO/CAM-FSHR mice. Data were collected for 2-3 weeks after first day of vaginal opening. Statistical analysis was conducted via 1-way ANOVA with Dunnett’s multiple comparison post hoc analysis. Statistical difference denoted by different letters, with a versus b *P* < .001. N/A represents mice that were acyclic, therefore cycle length could not be calculated.

Together, these data show that the LuRKO/CAM FSHR females demonstrated improvement in several reproductive parameters compared with LuRKO mice, including advanced follicle maturation, normalization of vaginal opening, mammary gland elongation, and increased uterine weight, all indicating enhanced estrogenic activity in the absence of LHR. This boosted follicular maturation is in line with our previous work showing accelerated follicle maturation in the CAM FSHR mice (18) and provides additional insight into how constitutive activation of FSHR can partially replace the function of LHR on antral follicle maturation to the preovulatory stage. In LuRKO/CAM FSHR males, CAM FSHR is able to induce androgen-dependent, Sertoli cell–expressed genes and thus can replace the androgen stimulus and rescue spermatogenesis (20). However, in females, intact LHR/LH signaling, or at least functional LHR expression, is ultimately required for ovulation and luteinization of follicle remnant. CAM FSHR cannot induce luteinization in absence of LHR, while in WT background luteinization was often observed, though with ovulation failure and “trapped” oocytes (Fig. 3C and (18)). Together this emphasizes the importance of accurate timing and regulation FSH and LH surges for ovulation and suggests that regulation of spermatogenesis is less sensitive to spatial/temporal regulation of gonadotrophin hormone receptor expression and signal pathway activation. As previously discussed, the physiological roles of LHR/FSHR heteromers have been postulated, with proposed roles in modulating signal specificity within the ovulatory follicle (47-49). Although enhanced ligand-independent cAMP signaling has been previously described for this CAM FSHR (18), the constitutive activation of additional FSH/FSHR pathways such as PI3 kinase/AKT, β-arrestin, and ERK-MAPK, and Gαq mediated Ca^2+^ and PKC signaling in ovaries, as well as in testes, remains unknown.

Ovarian function of the CAM FSHR/LuRKO mice agrees with our previous findings on FSH-treated LuRKO mice (46), where FSH stimulation was unable to promote follicular maturation beyond the antral stage. This contrasts with previous studies with hypophysectomized rats and mice, where ovulation and luteinization could be induced by treatment with recombinant FSH without LH (50-52). It proposes the necessity of intact LHR expression, even without ligand, for ovulation. One possibility is that this response requires LHR/FSHR heterodimerization to transduce the complete FSH signal.

## Conclusions

This study has utilized 3 approaches to interrogate the mode and nature of LHR signaling in modulating ovarian function. It suggests the importance of the spatial–temporal changes in LHR expression and the requirement for LH-dependent pleiotropic signaling for mediating aspects of postnatal sexual development and ultimately ovulation. These data also suggest important sexual dimorphism in the relative importance of intact LHR signaling for female gonadal function, that is sensitive to receptor number and cannot be overcome by promiscuous G protein-coupled receptor signaling and enhanced cAMP production. Yet, outstanding questions remain surrounding the functional relevance of LHR homomerization and LHR/FSHR heterodimerization within the ovary, questions that will no doubt be answered by the rapidly evolving gene editing approaches that are now available. Additionally, although LHR is essential for ovarian function, the postulated extragonadal roles of gonadotropic hormones in pregnancy and relevance of gonadotropin receptors for mediating these roles remains to be determined.

## Data Availability

Some or all data generated or analyzed during this study are included in this published article or in the data repositories listed in References.

## Abbreviations

ANOVA: analysis of variance
BAC: bacterial artificial chromosome
CAM: constitutively activating mutant
FSH: follicle-stimulating hormone
hCG: human chorionic gonadotropin
LH: luteinizing hormone
LHR: luteinizing hormone receptor
LuRKO: LHR knockout
qPCR: quantitative polymerase chain reaction
RT: reverse transcription
WT: wild type

## Additional Information

***Disclosures:*** The authors have nothing to disclose.
